# Absence of polysialylated NCAM is an unfavorable prognostic phenotype for advanced stage neuroblastoma

**DOI:** 10.1186/1471-2407-9-57

**Published:** 2009-02-17

**Authors:** Miikka Korja, Anne Jokilammi, Toivo T Salmi, Hannu Kalimo, Tarja-Terttu Pelliniemi, Jorma Isola, Immo Rantala, Hannu Haapasalo, Jukka Finne

**Affiliations:** 1 Department of Medical Biochemistry and Genetics, University of Turku, Turku, Finland; 2 Department of Pediatrics, Turku University Hospital, Turku, Turku, Finland; 3 Department of Pathology, Turku University Hospital, Turku, Turku, Finland; 4 Department of Clinical Chemistry, Turku University Hospital, Turku, Finland; 5 Laboratory of Cancer Biology, Institute of Medical Technology, University of Tampere and Tampere University Hospital, Tampere, Finland; 6 Department of Pathology, Tampere University Hospital, Tampere, Finland

## Abstract

**Background:**

The expression of a neural crest stem cell marker, polysialic acid (polySia), and its main carrier, neural cell adhesion molecule (NCAM), have been detected in some malignant tumors with high metastatic activity and unfavorable prognosis, but the diagnostic and prognostic value of polySia-NCAM in neuroblastoma is unclear.

**Methods:**

A tumor tissue microarray (TMA) of 36 paraffin-embedded neuroblastoma samples was utilized to detect polySia-NCAM expression with a polySia-binding fluorescent fusion protein, and polySia-NCAM expression was compared with clinical stage, age, *MYCN *amplification status, histology (INPC), and proliferation index (PI).

**Results:**

PolySia-NCAM-positive neuroblastoma patients had more often metastases at diagnosis, and polySia-NCAM expression associated with advanced disease (*P *= 0.047). Most interestingly, absence of polySia-NCAM-expressing tumor cells in TMA samples, however, was a strong unfavorable prognostic factor for overall survival in advanced disease (*P *= 0.0004), especially when *MYCN *was not amplified. PolySia-NCAM-expressing bone marrow metastases were easily detected in smears, aspirates and biopsies.

**Conclusion:**

PolySia-NCAM appears to be a new clinically significant molecular marker in neuroblastoma, hopefully with additional value in neuroblastoma risk stratification.

## Background

Neuroblastoma is a highly malignant and metastatic childhood cancer, which originates from the neural crest. Bone marrow is the most common metastatic site of neuroblastomas; up to 60% of children have bone marrow involvement at presentation [1]. Especially children older than one year at diagnosis have metastatic disease, and the overall 5-year event-free survival of stage 4 patients is only 16% [2]. In general, patients classified as high-risk for disease relapse have overall survival rates less than 40% despite intensive multimodal therapy. Currently, prognostic and therapeutic risk stratification is based primarily on age (dichotomized around 18 months), stage, and *MYCN*-amplification status.

Neural cell adhesion molecule (NCAM) is a cell surface molecule, which is expressed in the nervous system and in various cell types, such as natural killer cells (NK cells), cardiomyocytes, and neuroendocrine cells. NCAM expression has also been detected in many malignancies, *e.g. *in neuroblastoma, rhabdomyosarcoma, non-small cell lung carcinoma (NSCLC), small cell lung cancer (SCLC), brain tumors, myelomas and acute myeloid leukemia [3-9]. Although NCAM expression is not solely restricted to malignant cells, high-level expression of NCAM in a variety of malignancies makes an anti-NCAM approach attractive for cancer therapy. *In vivo *and *in vitro *experiments with huN901-DM1, a humanized variant of the murine monoclonal anti-NCAM antibody [10], support the idea of utilizing therapeutic anti-NCAM antibodies [8,11]. Currently, huN901-DM1-based anti-NCAM treatments of small cell lung cancer, NCAM-positive solid tumors and multiple myeloma are in phase I/II clinical studies http://www.immunogen.com/wt/page/IMGN901b.

Polysialic acid (polySia) is a linear α-2–8-linked sialic acid polymer, which is covalently attached to the NCAM molecule. PolySia is abundantly expressed in neural tissue during embryonic development, but progressively downregulated during maturation and differentiation [12,13]. Although polySia is virtually absent in the majority of adult tissues, it is reexpressed during the progression of some metastasis-prone malignant tumors, such as neuroblastoma, rhabdomyosarcoma, NSCLC and SCLC [14-18]. In these tumors, polysialylation appears to increase the metastatic behavior [16,18-20]. Moreover, it has been reported that polySia increases the motility of SCLC cells, and allows the cancer cells to detach from the primary tumor, thus causing the formation of metastatic foci [21]. *In vitro*, a significant reduction in neuroblastoma cell proliferation has been demonstrated after removal of polySia from the cell surface [22,23].

We conducted the search for polySia-NCAM and NCAM by utilizing a tumor tissue microarray (TMA) of the 36 paraffin-embedded neuroblastoma samples, as described earlier [24]. The expression of the oncodevelopmental antigen polySia and its main carrier NCAM was compared with the age, stage, *MYCN*-amplification status, histology according to the International Neuroblastoma Pathology Classification (INPC) [25] and proliferation index (PI).

## Methods

### Human tissue and tumor classifications

The study material consisted of a total of 37 paraffin-embedded neuroblastoma samples from 37 patients, which have been described in more detail previously [24,26]. Because of sample detachment, the expression pattern of polySia and NCAM could not be analyzed in one TMA sample. Therefore, the overall number of TMA samples was 36, of which 31 were primary neuroblastoma samples taken before any treatments. For histological typing and grading, the INPC system was applied [25]. The clinical stage of 22 cases could not be evaluated according to the International Neuroblastoma Staging System (INSS) [27], because these tumors were diagnosed before 1993, when protocols evaluating dissemination status were heterogeneous. Therefore, dissemination status was evaluated on the basis of medical records, and the clinical stage of the disease was divided into four categories: 1) local tumor, extirpation (completely excised); 2) local tumor with regional lymph node involvement; 3) distant metastases; 4) tumors that met INSS Stage 4S definition [27].

A total of 19 paraffin-embedded bone marrow samples (range 1–7 samples per patient; median two samples per patient) from six patients was available. The original histopathological analysis of the samples revealed two certain and one uncertain bone marrow involvements. These three samples had been taken from the same patient (diagnosed in 2001) at different time points. The primary tumor was undifferentiated neuroblastoma (stage 4) with unfavorable histology (INPC).

### Immunostaining of molecular markers and detection of MYCN amplification in TMAs

TMAs of neuroblastomas were constructed as described earlier [24]. Tumor TMA slides, bone marrow core biopsy samples on slides, and bone marrow aspiration smears were used for conventional immunostainings. Proliferation indices (PIs) with anti-Ki-67 antibody were detected as described earlier [24]. PolySia-binding fluorescent fusion protein (EndoNA2-GFP) at a concentration of 10 μg/ml was used for polySia detection as described earlier [28]. Mouse anti-human NCAM antibody (123C3) at a concentration of 4 μg/ml (Santa Cruz Biotechnology, Santa Cruz, CA) was used as a primary antibody. Immunohistochemical incubations were done overnight at 4°C. In immunofluorescence, Alexa Fluor 594 chicken anti-mouse secondary antibodies (Molecular Probes, Eugene, OR) were used, and slides were mounted with Immu-Mount (Shandon, USA). The *MYCN *copy number in tumor TMAs of neuroblastomas was quantified by CISH analysis with a digoxigenin-labeled probe complementary to the *MYCN *gene (Spot-Light N-Myc Probe, Zymed, South San Francisco, CA) as previously described [24].

### Bone marrow smears and flow cytometry

Bone marrow samples were prepared from an aspirate, obtained from an 18-year-old man, whose bone marrow was examined for nonmalignant anemia. For bone marrow smears, bone marrow cells were mixed with *in vitro *cultured human neuroblastoma SH-SY5Y cells (0, 50 and 100%), and the EndoNA2-GFP fusion protein [28] at a concentration of 10 μg/ml was used for staining the smears. For flow cytometry, bone marrow cells (100 μl, 3.5 × 10^6^) and polySia-NCAM-positive SH-SY5Y cells (100 μl, 3.5 × 10^6^) were labeled with the EndoNA2-GFP (10 μg/ml) fusion protein [28] for 20 minutes at room temperature. Red cells were lysed with FACS lysis buffer (Becton-Dickinson, NJ, USA). The cell suspensions were analyzed with a FACSCalibur flow cytometer (Becton-Dickinson).

### Confocal microscopy

We performed confocal scanning microscopy for the TMA of the 36 paraffin-embedded neuroblastoma samples using a Leica TCS SP MP confocal microscope equipped with a Spectra-Physics Tsunami Ti-sapphire laser and Leica confocal software. Sections were examined at two excitation wavelengths: 488 nm for polySia-binding fusion protein and 546 nm for fluorescent secondary antibodies.

### Statistical analyses

Differences between two groups in the categorical data were analyzed by means of the Pearson chi-square test. Overall survival analysis was computed using the Kaplan-Meier method, and the differences between the curves were compared using the log-rank test. Differences in mean values between multiple groups were analyzed using one-way Anova. All statistical analyses were performed with SPSS 16.0 for Macintosh, and *P *values of < 0.05 were considered statistically significant.

## Results

### PolySia and NCAM expression in paraffin-embedded neuroblastoma samples

**Figure 1:**
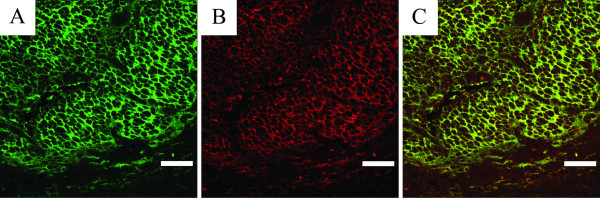
**The staining pattern of a primary neuroblastoma**. *A*, expression of polySia-NCAM detected with the EndoNA2-GFP fusion protein. *B*, NCAM expression (stained with Alexa Fluor 594) of the same location. *C*, overlay image of *A *and *B *identifying colocalized expression of polySia-NCAM and NCAM. Scale bar, 40 μm.

PolySia expression proved to be positive in 17 (54%) out of 31 patients, whose neuroblastoma samples were taken from primary tumors before any treatments (*i.e. *no treatment effect on antigen expression). An example of the staining pattern of polySia-NCAM is given in Figure 1. PolySia-specific immunofluorescence was confirmed with pretreatment of neuroblastoma samples with endosialidase, which abolished polySia-positive immunofluorescent signals. All three metastatic neuroblastoma samples from three patients were polySia- and NCAM-positive. The unselected and overall proportion of patients with polySia-positive neuroblastomas was 21 (58%) out of 36. In our analysis series, there were no polySia-expressing tumors, which did not show concomitant and colocalized NCAM expression. NCAM is the carrier of cell surface polySia: colocalization of polySia-NCAM and NCAM is shown in Figure 1.

NCAM expression was positive in 20 (65%) out of 31 primary neuroblastoma samples, which were taken before any treatments. The NCAM staining pattern is shown in Figure 1. A negative NCAM staining control was obtained by omission of the primary antibody. Three out of 20 NCAM-positive primary neuroblastomas did not express polySia-NCAM. The unselected and overall proportion of patients with NCAM-positive tumors was 24 (67%) out of 36.

The bone marrow disease was previously evaluated in 14 out of 31 patients with untreated primary neuroblastomas, and 11 of these 14 patients had been reported (on the basis of medical records) to have bone marrow metastases at diagnosis. PolySia-NCAM and NCAM expression in untreated primary tumor samples (not metastatic samples from regional lymph nodes or distant organs) was positive in 9 out of 11 patients with bone marrow disease. One out of three patients without bone marrow involvement had a polySia-NCAM- and NCAM-positive primary neuroblastoma.

### Association of polySia-NCAM and NCAM expression with clinical parameters (age and stage)

Patients' age did not associate with polySia-NCAM or NCAM expression, not even when groups were dichotomized at age of 12 and 18 months (Table 1). PolySia-NCAM expression in neuroblastomas did associate with clinical stage, as patients with polySia-NCAM-expressing primary tumors had advanced (*i.e. *metastatic) disease at diagnosis (*P *= 0.047) (Table 1). The association between NCAM expression and clinical stage was nearly significant (*P *= 0.053) (Table 1).

**Table 1:** Association of polySia-NCAM and NCAM expression of untreated primary neuroblastomas with clinical variables.

Variable	PolySia-NCAM-positive (17)	PolySia-NCAM-negative (14)	P value	NCAM positive (20)	NCAM negative (11)	P value
MEAN AGE (31)	2.9 ± 3.3	4.6 ± 5.0	0.258	3.4 ± 3.9	4.1 ± 4.7	0.683
AGE (31)			0.613			0.890
< 1.0 year (8)	5	3		5	3	
> 1.0 year (23)	12	11		15	8	
			0.465			0.939
< 1.5 yrs (11)	7	4		7	4	
> 1.5 yrs (20)	10	10		13	7	
CLINICAL STAGE (31)			0.047			0.053
LD, extirpation (7)	1	6		2	5	
LD with RLNI (4)	3	1		3	1	
Distant metastases (19)	13	6		15	4	
INSS 4S (1)	0	1			1	

Number of patients in each variable group is shown in parenthesis. *P*-values are measured for differences between categorical variables (polySia-NCAM or NCAM expression status). Abbreviations: LD = local disease; RLNI = regional lymph node involvement; BM = bone marrow; INSS = International Neuroblastoma Staging System

### Association of polySia-NCAM and NCAM expression with histological parameters (INPC, proliferation index [PI] and MYCN amplification)

Analysis of the correlation between polySia-NCAM expression and tumor differentiation (INPC) revealed that polySia-NCAM and NCAM were expressed more frequently in undifferentiated and in poorly differentiating than in differentiated neuroblastomas, but the *P*-value did not reach significance (*P *= 0.623 for polySia-NCAM, and *P *= 0.165 for NCAM) (Table 2). PolySia-NCAM- and NCAM-expressing primary tumors showed higher PIs than polySia-NCAM- and NCAM-negative tumors (*P *= 0.011 for polySia-NCAM, and *P *= 0.001 for NCAM) (Table 2). There was no association between polySia-NCAM or NCAM expression and *MYCN *amplification status (Table 2). All patients with *MYCN*-amplified neuroblastomas (seven patients) had stage 4 disease (*i.e. *distant metastases) at diagnosis.

**Table 2:** Association of polySia-NCAM and NCAM expression of untreated primary neuroblastomas with histological variables.

Variable	PolySia-NCAM-positive (17)	PolySia-NCAM-negative (14)	P value	NCAM positive (20)	NCAM negative (11)	P value
INPC (30*)		*	0.623		*	0.165
Undifferentiated (12)	8	4		10	2	
Poorly differentiated (6)	4	2		5	1	
Differentiating (9)	4	5		4	5	
Ganglioneuroblastoma (2)	1	1		1	1	
Ganglioneuroma (1)		1			1	
MEAN PI (31)	36.0 ± 17.6	19.9 ± 14.7	0.011	36.1 ± 16.6	15.3 ± 12.3	0.001
MYCN (31)			0.316			0.183
nonamplified (24)	12	12		14	10	
amplified (7)	5	2		6	1	

Number of patients in each group is shown in parenthesis. *P*-values are measured for differences between categorical variables (polySia-NCAM or NCAM expression status). *An intracranial neuroblastoma was excluded from the INPC classification. Abbreviations: INPC = International Neuroblastoma Pathology Classification; PI = proliferation index.

### Survival analyses

**Figure 2:**
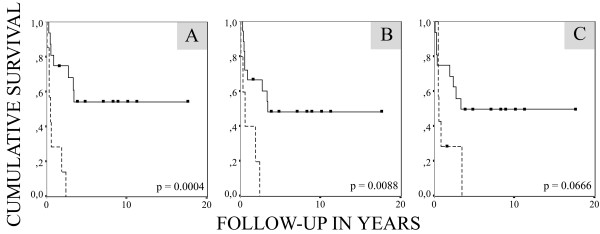
**Kaplan-Meier survival curve for 23 patients in the advanced disease group, including patients having LC with regional lymph node involvement (4 patients) and patients with distant metastases (19 patients), stratified by polySia-NCAM expression (*A*), NCAM expression (*B*), and *MYCN *amplification status (*C*)**. The solid line represents polySia-NCAM-positive patients (16 patients), and the broken line represents polySia-NCAM-negative patients (7 patients).

Negative polySia-NCAM expression was a strong unfavorable predictor of overall survival in advanced disease, as all seven patients with negative polySia-NCAM expression and regional lymph node involvement or distant metastases died during the follow-up time (Fig. 2) (*P *= 0.0004). Negative NCAM expression was also an unfavorable predictor of outcome (*P *= 0.0088) in the same advanced disease group (Fig. 2). When the prognostic value of polySia-NCAM and NCAM were evaluated solely for the 19 patients with distant metastases, they still reached significant P-values (*P *= 0.0019 and *P *= 0.0240, respectively). In comparison to the prognostic value of *MYCN *amplification (not significant, *P *= 0.0666), polySia-NCAM and NCAM were stronger predictors of unfavorable outcome in patients with advanced disease (Fig. 2). The overall prognostic significance of *MYCN *amplification in the hotspot analysis has been presented previously [24].

### Detection of polySia-NCAM-positive neuroblastic cells in bone marrow with immunofluorescence and flow cytometry

**Figure 3:**
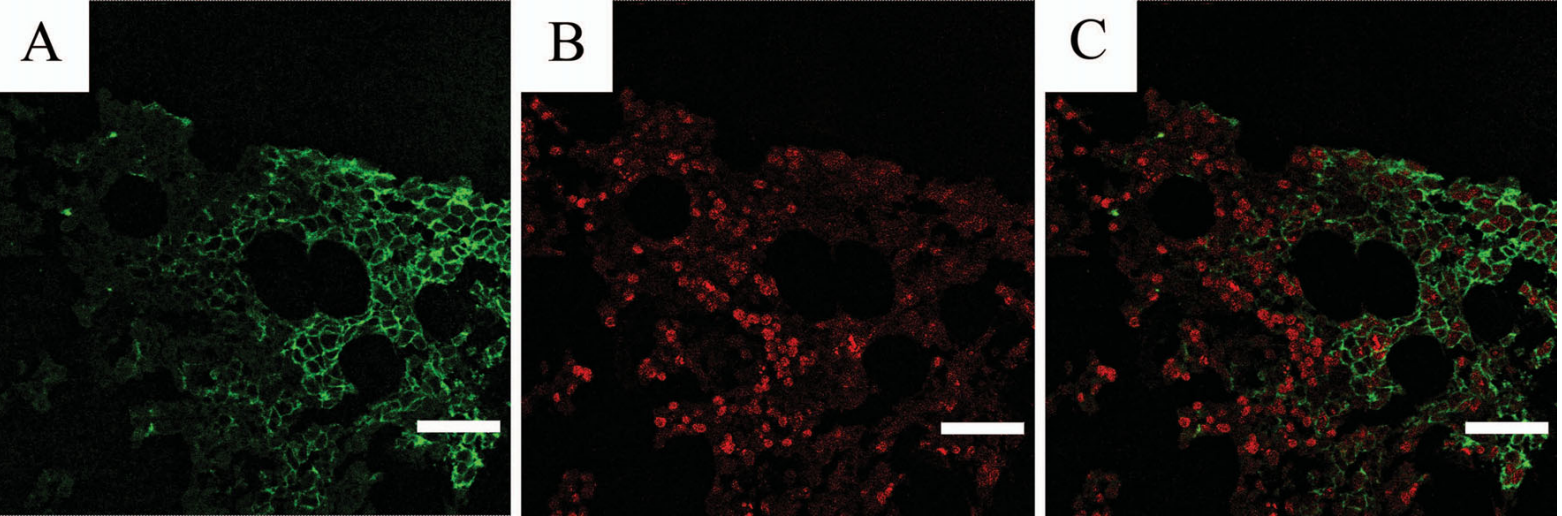
**PolySia-NCAM-positive neuroblastoma cells in a bone marrow sample were double-labeled with EndoNA2-GFP (*A*), and anti-Ki-67 antibody (stained with Alexa Fluor 594) (*B*)**. *C*, overlay image of *A *and *B *showing proliferating cells in context of polySia-NCAM expression. Scale bar, 40 μm.

To investigate whether metastatic neuroblastoma cells in bone marrow express polySia-NCAM, like their polySia-NCAM positive primary tumor foci, 19 paraffin-embedded bone marrow biopsies from six different patients were labeled with the fluorescent fusion protein. PolySia-NCAM-positive tumor cell clusters, as shown in Figure 3, were found in two different bone marrow samples, taken at different time points from the same patient with a polySia-NCAM positive primary tumor. Contrary to the primary tumors, polySia-NCAM positive bone marrow neuroblastoma cells appeared to be in a non-proliferative state, when samples were double-labeled with anti-Ki-67 antibody (Fig. 3).

Fresh bone marrow cells and *in vitro *cultured polySia-NCAM-positive SH-SY5Y neuroblastic cells were mixed together in different ratios, and the mixed cell suspensions were labeled with the polySia-binding fluorescent fusion protein. Interestingly, smears with added polySia-NCAM-positive SH-SY5Y cells appeared in cell clusters, like in fixed bone marrow biopsy samples, whereas smears without added SH-SY5Y cells did not show any cell clustering. Normal bone marrow samples were considered polySia-NCAM-negative, even though they contained a few single cells (approximately 1–3 individual cells per microscopic field), which were polySia-NCAM-positive, but significantly smaller in size than polySia-NCAM-positive tumor cell clusters.

**Figure 4:**
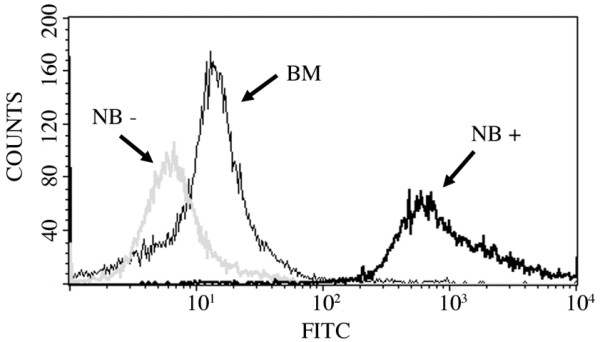
**Flow cytometry analysis of cultured polySia-NCAM-positive SH-SY5Y cells (NB +) and normal bone marrow cells (BM) stained with polySia-binding EndoNA2-GFP fusion protein**. Unstained SH-SY5Y cells (NB-) served as negative control sample.

In evaluating the possible applicability of flow cytometry to differentiate bone marrow metastases (polySia-NCAM-positive neuroblastoma cells or cell clusters) from normal bone marrow cells, our results revealed that polySia-NCAM-positive tumor cells produce distinct fluorescence when compared to fusion protein-labeled normal bone marrow cells (Fig. 4). As we had no permission to take fresh bone marrow samples from neuroblastoma patients for research purposes, we had to use the above-described artificially created "stage 4 bone marrow samples" to test the applicability of polySia-based detection of neuroblastoma cells in fresh samples.

## Discussion

PolySia-NCAM expression was proved to associate with clinical stage: patients with polySia-NCAM-expressing primary neuroblastomas were shown to have an advanced disease at diagnosis (*P *= 0.047). Most interestingly, positive polySia-NCAM expression is a strong prognostic factor for favorable overall survival (*P *= 0.0004) in advanced disease, especially when the *MYCN *copy number is not amplified. Furthermore, the vast majority (9 out of 11) of bone marrow disseminated primary neuroblastomas had polySia-NCAM on their cell surfaces. Because neither polySia-NCAM nor NCAM expression associated with *MYCN *amplification (Table 2), which is a common feature in metastatic neuroblastoma, it appears that polySia-NCAM may be an independent marker for metastatic activity in neuroblastoma, like in rhabdomyosarcoma, NSCLC and SCLC [16,18,20]. In addition, the presence of polySia-NCAM on cell surfaces was shown to associate with increased neuroblastoma SH-SY5Y cell proliferation in an *in vitro *assay (results not shown) and, for the first time, *in vivo *(*P *= 0.011). Previously, polysialylation of NCAM has been shown to increase proliferation activity *in vitro *[22]. Increased cell proliferation may favor the metastatic activity of primary tumor cells. In general, both polySia-NCAM and NCAM are common antigens on untreated primary neuroblastomas, especially on immature (undifferentiated and poorly differentiated) neuroblastoma cells. Age, which is an important prognostic factor [2], does not seem to associate with the expression of these antigens. It has been suggested that immunohistochemically NCAM-negative NSCLC samples showing polySia expression, carry polySia on other molecules than NCAM [16,19]. On the basis of our results, NCAM appears to be the only carrier of polySia in neuroblastomas. As only three out of 20 NCAM-positive neuroblastomas were polySia-negative, the true independent prognostic value of NCAM expression could not be determined.

Current prognostic evaluations are based on clinical stage, age and *MYCN *amplification in neuroblastoma. These evaluations often overestimate the number of patients in need of chemotherapy, because the evaluation cannot define patients with stage 1–3 tumors that are capable of spontaneous or easily induced regression. The prognostic and therapeutic evaluation of stage 2–4 patients with the normal *MYCN *copy number is often challenging, too. According to our results, polySia-NCAM-expressing neuroblastoma patients with distant metastases (13 patients) survived significantly better (6 out of 13 alive, *P *= 0.0004) than polySia-NCAM-negative neuroblastoma patients with distant metastases (6 patients, all dead). Very interestingly, five out of seven dead patients in the favorable prognostic group had polySia-NCAM-positive primary tumors (a favorable factor) with *MYCN *amplification (an unfavorable factor). In other words, *MYCN* amplification seems to counteract the positive prognostic value of polySia-NCAM in advanced disease group. Even though current intensive multimodality treatment protocols may improve the overall survival of high-risk patients, it is unlikely that a difference in treatment protocols explains our result. In summary, patients with polySia-NCAM-negative a.) local neuroblastoma with involvement of regional lymph nodes (equal to INSS stage 2B-3 tumors), or b.) neuroblastoma with distant metastases (equal to INSS stage 4 tumors) have an unfavorable prognosis despite *MYCN *amplification status, whereas patients with polySia-NCAM-positive neuroblastomas (similar to INSS stage 2B-4 tumors) have a more favorable prognosis, especially when the *MYCN *copy number is normal. *In vitro *studies have shown that polySia increases the motility of SCLC cells, and allows the cancer cells to detach from the primary tumor, thus causing the formation of metastatic foci [21]. In conclusion, polySia-NCAM is considered to be a neural stem cell marker [29], and widely spread polySia-NCAM neuroblastoma cells (of neural crest origin) may be neural stem cell-like cells obviously harboring other than proliferative and mitotic stem cell characteristics, such as differentiation, which improves prognosis.

Bone marrow is the most important secondary organ for the detection of circulating neuroblastoma cells. Our results show that 9 out of 11 (82%) patients with bone marrow metastases at diagnosis had primary tumors expressing polySia-NCAM. We also detected polySia-NCAM-expressing neuroblastic cells in two paraffin-embedded bone marrow samples. Interestingly, these tumor cells had originated from a polySia-NCAM-positive primary neuroblastoma. Previously, bone marrow metastases in advanced stages of neuroblastoma were shown to be NCAM-positive, but the polysialylation of NCAM molecules was not determined [30]. PolySia-NCAM-expressing tumor cells in bone marrow grew in clusters, which is in accordance with previous *in vitro *results indicating that polySia-NCAM-expressing SH-SY5Y neuroblastic tumor cells never grow without contact with each other, even at low densities [23]. PolySia-NCAM-positive tumor cells in bone marrow rarely stained with anti-Ki-67 antibody. The low proliferation activity of polySia-NCAM-positive tumor cells in bone marrow might explain, to some extent, the relative resistance of bone marrow metastases to conventional chemotherapy. Very recently, it was presented that the transcript level of one of the two key enzymes responsible for polySia synthesis, sialyltransferase STX, is a highly prognostic outcome factor for neuroblastoma, if measured from bone marrow after cytotoxic treatments [19]. In that study, concomitant *MYCN *amplification may explain unfavorable prognosis in polySia-NCAM positive patients. However, if polySia-NCAM-positive neuroblastoma cells in bone marrow need to be treated or detected, the intensity and specificity of our recently developed polySia-detecting fusion protein [28] allows it to be used in the detection of metastatic disease in fresh bone marrow samples by flow cytometry, in addition to immunohistochemical identification of minimal residual disease in biopsies or smears. In summary, polySia-NCAM expression may favor neuroblastoma cell dissemination into bone marrow.

## Conclusion

A subpopulation of cells in untreated primary neuroblastomas contains adhesive characteristics, namely polySia-NCAM phenotype, which associates with proliferation activity and clinical stage. Moreover, polySia-NCAM appears to be a pathogenetically relevant marker predicting outcome, especially in advanced stage neuroblastoma, which to date has remained a therapeutical challenge. The specific and sensitive detection of polySia-NCAM-expressing primary and metastatic tumor cells may allow the development of new immunodiagnostic and immunotherapeutical approaches. A better immunohistochemical prediction of neuroblastoma behavior at diagnosis may help to avoid treatment failure in high-risk patients. Due to the limited number of patients, the presented results have to be interpreted with caution. Therefore, a larger study is warranted.

## Competing interests

The authors declare that they have no competing interests.

## Authors' contributions

MK drafted and revised the manuscript. MK and AJ carried out the immunohistochemical and confocal studies. AJ and T-TP were responsible for the flow cytometry experiments. TTS participated in the patient selection and manuscript drafting. HK and HH carried out pathological staging of the neuroblastoma samples. JI participated in the analysis of MYCN status. IR participated in the design of the immunohistochemical analyses. JF conceived of the study, and participated in its design and coordination and helped to draft the manuscript. All authors read and approved the final manuscript.

## Pre-publication history

The pre-publication history for this paper can be accessed here:

http://www.biomedcentral.com/1471-2407/9/57/prepub
